# Some sauropods raised their necks—evidence for high browsing in *Euhelopus zdanskyi*

**DOI:** 10.1098/rsbl.2010.0359

**Published:** 2010-06-02

**Authors:** Andreas Christian

**Affiliations:** Universität Flensburg, Institut für Biologie und ihre Didaktik, Auf dem Campus 1, 24943 Flensburg, Germany

**Keywords:** sauropod, dinosaur, neck, feeding, energy expenditures

## Abstract

A very long neck that is apparently suitable for feeding at great heights is a characteristic feature of most sauropod dinosaurs. Yet, it remains controversial whether any sauropods actually raised their necks high. Recently, strong physiological arguments have been put forward against the idea of high-browsing sauropods, because of the very high blood pressure that appears to be inevitable when the head is located several metres above the heart. For the sauropod *Euhelopus zdanskyi*, however, biomechanical evidence clearly indicates high browsing. Energy expenditure owing to high browsing is compared with energy costs for walking a distance. It is demonstrated for *Euhelopus* as well as for the much larger *Brachiosaurus* that despite an increase in the metabolic rate, high browsing was worthwhile for a sauropod if resources were far apart.

## 1. Introduction

Because of their extreme size, sauropods attract much interest from scientists of various disciplines. The largest sauropods might have almost reached biomechanical and physiological limits. Recent findings indicate fast growth and high metabolic rates in sauropods (Sander & Clauss 2008). Consequently, the rate of food intake must have been very high. A selective advantage of the usually very long sauropod neck for feeding appears unquestionable. Yet, the posture and the utilization of sauropod necks remain the focus of a long debate. The neck may have been used for increasing the horizontal feeding range (Martin 1987) or for high browsing (Bakker 1986; Paul 1988). Whereas most researchers agree on low browsing in some forms like *Diplodocus*, *Apatosaurus* (Stevens & Parrish 1999) and *Nigersaurus* (Sereno *et al*. 2007), the question remains open if any sauropod actually exploited resources at great heights.

The assumption of ecological niche partitioning among sauropods (Dodson 1990) with different species of the same habitat browsing at different heights appears reasonable and fits the observed variation in tooth and jaw morphology (Upchurch & Barrett 2000; Sereno & Wilson 2005). However, arguments have been put forward against the idea of high browsing in sauropods. According to Stevens & Parrish (1999, 2005), optimal articulation of the neck vertebrae and neck flexibility indicate a low neck position especially in sauropods with extremely long necks. However, Dzemski & Christian (2007); Christian & Dzemski (2007) and Taylor *et al*. (2009) refuted the hypothesis that the osteological neutral pose was commonly adopted in life. Strong physiological arguments against high browsing have been formulated by Seymour (2009*a*,*b*). Seymour (2009*a*) points out that high browsing results in high stress on the cardiovascular system because a very high blood pressure is required for supplying the brain with blood if the head is several metres above the heart. According to Seymour (2009*b*), energy expenditures due to a higher blood pressure increase greatly with feeding height, whereas maximum food intake decreases, so that high browsing is not worthwhile. In the light of these arguments, maintaining the concept of high-browsing sauropods requires strong evidence. Such evidence is given here for *Euhelopus zdanskyi* (Wiman 1929), a moderately sized sauropod with an excellently preserved neck skeleton (Wilson & Upchurch 2009). For the much larger *Brachiosaurus brancai*, biomechanical arguments also support the idea of high browsing (Christian & Dzemski 2007). Additional mechanisms like ‘neck hearts’ that might have enabled sauropods to increase the blood pressure in the head and neck independently from the body remain speculative. It will be demonstrated that even without such mechanisms high browsing was also worthwhile for *Brachiosaurus* if food sources were widely spaced.

## 2. Material and methods

Measurements of the skeletal dimensions of *Euhelopus zdanskyi* were taken from specimen PMU 233, exhibited at the University Uppsala, Sweden. Additional data were taken from description and illustrations by Wiman (1929). Data lacking due to damaged vertebrae were interpolated. Based on the dimensions of the neck skeleton, the mass distribution along the neck was reconstructed under the assumption of a low neck density owing to strong pneumatization (Henderson 2004, 2006; Wedel 2005, 2009; Wilson & Upchurch 2009; see electronic supplementary material). For different hypothetical neck postures, the stress in the intervertebral cartilage was calculated along the neck (Christian 2002; see electronic supplementary material). Habitual positions of the neck at rest are characterized by approximately constant stress values along the neck (Christian 2002). Body mass was assumed to equal 3.8 metric tons (Mazzetta *et al*. 2004). Energy expenditures were calculated from the literature (Schmidt-Nielsen 1984; Seymour 2009*b*; White *et al*. 2009; see electronic supplementary material).

## 3. Results

**Figure 1.**
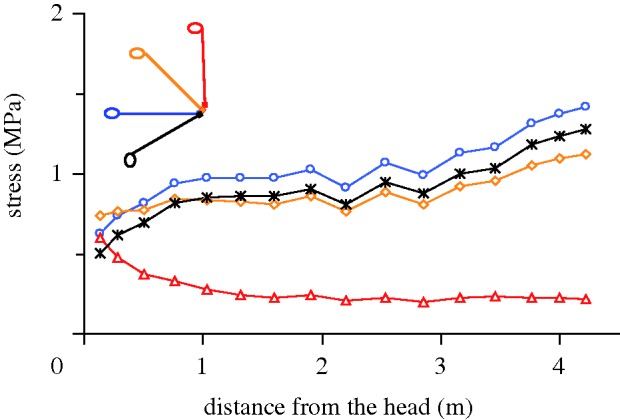
Stresses in the intervertebral joints along the neck of *Euhelopus zdanskyi* calculated for some hypothetical neck postures. Inclined postures yield the least variation in stress. Slightly lower values in the foremost neck section are usual because of additional muscle force for moving the head (Christian & Dzemski 2007). High values at the hindmost neck section indicate tensile structures that lay high above the vertebrae.

The estimated combined mass of neck and head of *Euhelopus* was about 210 kg (see electronic supplementary material). With a straight neck, the distance between the snout and the base of the neck was about 4.6 m. Nearly constant stress values in the intervertebral cartilage along the neck were only obtained in nearly straight neck poses with an angle between the neck and the horizontal of between 40° and 50°. Taking errors into account, especially in the estimated distribution of the neck mass, a slightly lower resting position of the neck is possible, but the results are neither in accordance with a fully vertical nor with a horizontal position of the neck (figure 1). Curved postures were also tested but did not yield constant stress values. Neck flexibility appears to have been generally low, except for the most distal and proximal neck regions, where lateral as well as dorsoventral motions were less restricted than in the long midsection of the neck. The dorsal spines of the vertebrae are very low or even lacking at the neck–trunk transition, indicating an upward bend in the vertebral column and long muscles or tendons that lay well above the vertebrae in this region.

**Figure 2.**
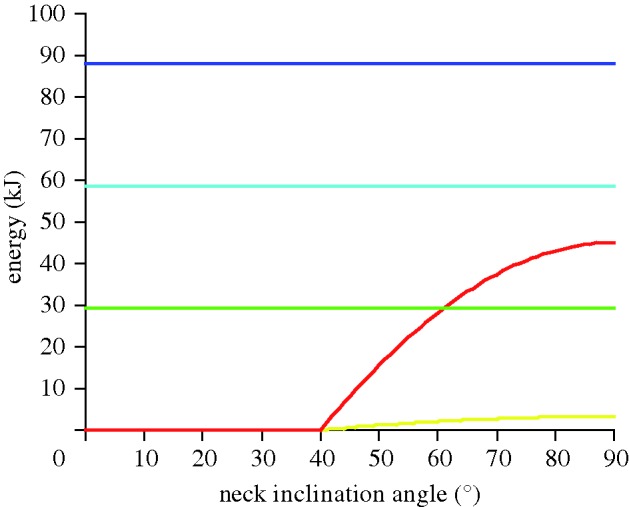
Energy expenditures for feeding at different heights for 5 min compared with the net energy costs for travelling different distances. The additional metabolic rate owing to an increased blood pressure is related to a resting posture of the neck with an inclination angle of 40° between the neck and the horizontal plane. Yellow line, raising the neck from the resting position; red line, keeping the neck for 5 min in position; green line, energy cost of transport: walking distance 10 m; blue line, energy cost of transport: walking distance 20 m; dark blue line, energy cost of transport: walking distance 30 m.

For *Euhelopus*, the energy costs for walking given distances are compared with the energy expenditure for raising the neck from a horizontal to an inclined position and the energy expenditures for maintaining a high blood pressure for 5 min during high browsing (figure 2). Mechanical work for raising the neck from an inclined to a vertical position is rather low and can be neglected if the head were raised only once during the time interval. In table 1, the energy costs for *Euhelopus* and *Brachiosaurus* for walking a distance of 100 m are compared with the energy expenditures for high browsing starting either from a horizontal or from a 40° inclined position of the neck. Raising the neck and feeding for the time intervals given in table 1 would have cost approximately the same as that of walking the distance of 100 m.

**Table 1.** Estimates of time intervals for browsing with a fully vertical neck that are energetically equivalent to walking a distance of 100 m: *T*_1_, time interval assuming a horizontal resting position of the neck; *T*_2_, time interval assuming an inclined (40°) resting position.

	*T*_1_ (min)	*T*_2_ (min)
*Euhelopus zdanskyi*	11.6	32.2
*Brachiosaurus brancai*	3.8	12.9

## 4. Discussion

The biomechanically reconstructed neck posture of *Euhelopus* is similar to that of a giraffe (*Giraffa camelopardalis*). As in giraffes, the neck of *Euhelopus* appears to have been kept rather straight, and changes in its position were mainly generated by flexion between the neck and the trunk as it is commonly observed in terrestrial vertebrates (Vidal *et al*. 1986).

Vertebrates that usually feed at low levels, like ostriches and camels, may also raise the head above the shoulders at rest. A low-browsing animal with a long neck, however, tends to limit vertical shifts of the centre of mass of the neck–head system by mainly moving the distal parts of the neck during browsing, whereas the height of the heavy hindmost neck section does not change very much. This feeding strategy can be observed among living vertebrates and has also been proposed for some sauropods, like *Diplodocus carnegii* (Dzemski & Christian 2007). It is expedient for high browsing to use the full length of the neck as giraffes do. For this feeding strategy, a rather rigid neck with reduced muscle mass is advantageous. In *Euhelopus*, the very long cervical ribs allowed transmission of forces in a controlled way over a long distance, thus shifting the muscle mass further back towards the trunk, as suggested by Christian & Dzemski (2007) for *Brachiosaurus*.

For *Euhelopus*, the static analysis and the flexibility pattern along the neck indicate browsing at medium and great heights. According to Seymour (2009*a*,*b*), high browsing is not worthwhile because of the additional energy cost for maintaining a high blood pressure combined with a decrease in food intake. This argument holds true only under the assumption of sufficient resources at low heights (Sander *et al*. 2009) and a homogeneous spatial distribution of food. If smaller sources of food were widely spaced, as it appears reasonable to assume for the environment in which many sauropods lived, a sauropod may have had two possibilities: raising the head for exploiting resources in great heights or walking a long distance to find food at lower heights. The energy expenditures illustrated in figure 2 and table 1 are only rough estimates. The general conclusion, however, is not affected by uncertainties in the data: *Euhelopus* and *Brachiosaurus* should have browsed for a few minutes with a vertical neck rather than travel a distance of several body lengths in order to obtain the same amount of food.

According to Seymour (2009*b*) raising a sauropod neck ‘costs more to get less’. Raising the neck, however, may have been less expensive for a sauropod like *Euhelopus* or *Brachiosaurus* than walking a long distance. During a food shortage, raising the neck was probably even essential for surviving: it is better to get little than nothing at all.

## References

[RSBL20100359C1] Bakker R. 1986 The dinosaur heresies. New York, NY: William Morrow

[RSBL20100359C2] Christian A. 2002 Neck posture and overall body design in sauropods. Mitteilungen des Museums für Naturkunde Berlin, Geowissenschaftliche Reihe 5, 269–279

[RSBL20100359C3] Christian A. Dzemski G. 2007 Reconstruction of the cervical skeleton posture of *Brachiosaurus brancai* Janensch, 1914 by an analysis of the intervertebral stress along the neck and a comparison with the results of different approaches. Fossil Rec. 10, 37–48

[RSBL20100359C4] Dodson P. 1990 Sauropod paleoecology. In The Dinosauria (eds Weishampel D. B. Dodson P. Osmólska H.), pp. 402–407 Berkeley, CA: University of California Press

[RSBL20100359C5] Dzemski G. Christian A. 2007 Flexibility along the neck of the ostrich (*Struthio camelus*) and consequences for the reconstruction of dinosaurs with extreme neck length. J. Morphol. 268, 701–714 (doi:10.1002/jmor.10542) 17514722 10.1002/jmor.10542

[RSBL20100359C6] Henderson D. M. 2004 Tipsy punters: sauropod dinosaur pneumaticity, buoyancy and aquatic habits. Proc. R. Soc. Lond. B (suppl.) 271, 180–183 (doi:10.1098/rspb.2003.0136) 10.1098/rsbl.2003.0136 PMC1810024 15252977

[RSBL20100359C7] Henderson D. M. 2006 Burly gaits: centers of mass, stability, and the trackways of sauropod dinosaurs. J. Vertebr. Paleontol. 26, 907–921 (doi:10.1671/0272-4634(2006)26[907:BGCOMS]2.0.CO;2)

[RSBL20100359C8] Mazzetta G. V. Christiansen P. Farina R. A. 2004 Giants and bizarres: body size of some southern South American cretaceous dinosaurs. Historic. Biol. 16, 71–83

[RSBL20100359C9] Martin J. 1987 Mobility and feeding of *Cetiosaurus* (Saurischia: Sauropoda)—why the long neck? In 4th Symp. Mesozoic Terrestrial Ecosystems (eds Curry P. J. Koster E. H.), pp. 154–159 Drumheller, Canada: Tyrell Museum of Paleontology

[RSBL20100359C10] Paul G. S. 1988 The brachiosaur giants of the Morrison and Tendaguru with a description of a new subgenus, *Giraffatitan*, and a comparison of the word's largest dinosaurs. Hunteria 2, 1–14

[RSBL20100359C11] Sander P. M. Clauss M. 2008 Sauropod gigantism. Science 322, 200–201 (doi:10.1126/science.1160904) 18845734 10.1126/science.1160904

[RSBL20100359C12] Sander P. M. Christian A. Gee C. T. 2009 Response to sauropods kept their necks down. Science 323, 167119325098

[RSBL20100359C13] Schmidt-Nielsen K. 1984 In Scaling—why is animal size so important. Cambridge, USA: Cambridge University Press

[RSBL20100359C14] Sereno P. C. Wilson J. A. 2005 Structure and evolution of a sauropod tooth battery. In The sauropods: evolution and paleobiology (eds Wilson J. A. Curry-Rogers K.), pp. 157–177 Berkeley, CA: University of California Press

[RSBL20100359C15] Sereno P. C. Wilson J. A. Witmer L. M. Whitlock J. A. Maga A. Ide O. Rowe T. A. 2007 Structural extremes in a cretaceous dinosaur. PLoS ONE 2, e1230 (doi:10.1371/journal.pone.0001230) 18030355 10.1371/journal.pone.0001230 PMC2077925

[RSBL20100359C16] Seymour R. S. 2009a Sauropods kept their heads down. Science 323, 1671 (doi:10.1126/science.323.5922.1671) 19325098 10.1126/science.323.5922.1671

[RSBL20100359C17] Seymour R. S. 2009b Raising the sauropod neck: it costs more to get less. Biol. Lett. 5, 317–319 (doi:10.1098/rsbl.2009.0096) 19364714 10.1098/rsbl.2009.0096 PMC2679936

[RSBL20100359C18] Stevens K. A. Parrish M. J. 1999 Neck posture and feeding habits of two Jurassic sauropod dinosaurs. Science 284, 798–800 (doi:10.1126/science.284.5415.798) 10221910 10.1126/science.284.5415.798

[RSBL20100359C19] Stevens K. A. Parrish M. J. 2005 Digital reconstructions of sauropod dinosaurs and implications for feeding. In The sauropods: evolution and paleobiology (eds Wilson J. A. Curry-Rogers K.), pp. 178–200 Berkeley, CA: University of California Press

[RSBL20100359C20] Taylor M. P. Wedel M. J. Naish D. 2009 Head and neck posture in sauropod dinosaurs inferred from extant animals. Acta Palaeontol. Pol. 54, 213–220 (doi:10.4202/app.2009.0007)

[RSBL20100359C21] Upchurch P. Barrett P. M. 2000 The evolution of sauropod feeding mechanisms. In Evolution of herbivory in terrestrial vertebrates: perspectives from the fossil record (ed. Sues H.-D.), pp. 79–122 Cambridge, UK: Cambridge University Press

[RSBL20100359C22] Vidal P. P. Graf W. Berthoz A. 1986 The orientation of the cervical vertebral column in unrestrained awake animals. Exp. Brain Res. 61, 549–559 3082659 10.1007/BF00237580

[RSBL20100359C23] Wedel M. J. 2005 Postcranial skeletal pneumaticity in sauropods and its implications for mass estimates. In The sauropods: evolution and paleobiology (eds Wilson J. A. Curry-Rogers K.), pp. 201–228 Berkeley, CA: University of California Press

[RSBL20100359C24] Wedel M. J. 2009 Evidence for bird-like air sacs in saurischian dinosaurs. J. Exp. Zool. 311A, 611–628 10.1002/jez.513 19204909

[RSBL20100359C25] White C. R. Blackburn T. M. Seymour R. S. 2009 Phylogenetically informed analysis of the allometry of mammalian basal metabolic rate supports neither geometric nor quarter-power scaling. Evolution 63, 2658–2667 (doi:10.1111/j.1558-5646.2009.00747.x) 19519636 10.1111/j.1558-5646.2009.00747.x

[RSBL20100359C26] Wilson J. A. Upchurch P. 2009 Redescription and reassessment of the phylogenetic affinities of *Euhelopus zdanskyi* (Dinosauria: Sauropoda) from the Early Cretaceous of China. J. Syst. Palaeontol. 7, 199–239

[RSBL20100359C27] Wiman C. 1929 Die Kreide-Dinosaurier aus Shantung. Palaeontol. Sin. (Ser. C) 6, 1–67

